# Glioblastoma: Is There Any Blood Biomarker with True Clinical Relevance?

**DOI:** 10.3390/ijms21165809

**Published:** 2020-08-13

**Authors:** Paulo Linhares, Bruno Carvalho, Rui Vaz, Bruno M. Costa

**Affiliations:** 1 Neurosurgery Department, Centro Hospitalar São João, Alameda Prof Hernani Monteiro, 4200–319 Porto, Portugal; paulojlinhares@yahoo.com (P.L.); ruimcvaz@gmail.com (R.V.); 2 Clinical Neurosciences and Mental Health Department, Faculty of Medicine, University of Oporto, Alameda Prof. Hernâni Monteiro, 4200-319 Porto, Portugal; 3 Life and Health Sciences Research Institute (ICVS), School of Medicine, University of Minho, Campus de Gualtar, 4710-057 Braga, Portugal; bfmcosta@med.uminho.pt; 4 ICVS/3B’s—PT Government Associate Laboratory, Braga/Guimarães, 4710-057 Braga, Portugal

**Keywords:** glioblastoma, biomarkers, prognosis, diagnosis

## Abstract

Glioblastoma (GBM) is the most frequent malignant primary brain tumor in adults, characterized by a highly aggressive, inflammatory and angiogenic phenotype. It is a remarkably heterogeneous tumor at several levels, including histopathologically, radiographically and genetically. The 2016 update of the WHO Classification of Tumours of the Central Nervous System highlighted molecular parameters as paramount features for the diagnosis, namely *IDH1/2* mutations that distinguish primary and secondary GBM. An ideal biomarker is a molecule that can be detected/quantified through simple non- or minimally invasive methods with the potential to assess cancer risk; promote early diagnosis; increase grading accuracy; and monitor disease evolution and treatment response, as well as fundamentally being restricted to one aspect. Blood-based biomarkers are particularly attractive due to their easy access and have been widely used for various cancer types. A number of serum biomarkers with multiple utilities for glioma have been reported that could classify glioma grades more precisely and provide prognostic value among these patients. At present, screening for gliomas has no clinical relevance. This is because of the low incidence, the lack of sensitive biomarkers in plasma, and the observation that gliomas may develop apparently de novo within few weeks or months. To the best of our knowledge, there is no routine use of a serum biomarker for clinical follow-up. The purpose of this paper is to review the serum biomarkers described in the literature related to glioblastoma and their possible relationship with clinical features.

## 1. Introduction

Glioblastomas (GBMs) are the most frequent primary malignant brain tumor, accounting for approximately 15% of all intracranial neoplasms and 45–50% of all primary malignant brain tumors [1]. The clinical behavior of individual tumors of a specific histopathological entity may differ substantially. Thus, additional markers are needed for a more refined and objective glioma classification, a better prediction of prognosis, and tailored therapeutic decision-making [2]. The revised 2016 World Health Organization (WHO) classification of tumors of the central nervous system recognizes the molecular evaluation as critical for the classification, since the mutation status of the *IDH1* and *IDH2* and the codeletion of 1p/19q are crucial for diagnosis, and the *TERT* promoter gene mutations and *ATRX* alterations are associated with prognosis. However, other molecular alterations may be associated with diagnosis, prognosis, and glioma risk.

A “biomarker” (biological marker) can be defined as “a characteristic that is objectively measured and evaluated as an indicator of normal biological processes, pathogenic processes, or pharmacological responses to a therapeutic intervention” [3]. The critical hallmark of a biomarker is to be highly sensitive and specific in providing information relevant for diagnosis, prognosis, or therapy [4]. Biomarkers should have the potential to identify cancer patients at an early stage, select patients who are most likely to benefit from therapy, and to guide the development of more effective agents [5]. The most useful tumor marker should possess high specificity and sensitivity to the tumor, and it should be unique.

GBMs show complex chromosomal and genetic alterations that lead to inactivation of various tumor suppressor genes, as well as aberrant activation of proto-oncogenes [2]. Glioblastoma *IDH*-wildtype lacks *IDH1/2* mutations and is considered primary glioblastoma. The typical genetic alterations of these tumors include *TERT* promoter mutations, homozygous deletion of *CDKN2A/CDKN2B*, loss of chromosomes 10p and 10q, *EGFR* alterations, *PTEN*, *TP53* and *PI3K* mutations [1]. Glioblastomas *IDH*-mutant show mutations in either the *IDH1* or *IDH2* gene and are characterized by frequent mutations in the *TP53* and *ATRX* genes, loss of 10q [1], and lack *EGFR* amplification [2]. They are mostly secondary, derived from lower-grade lesions. Often extensive tumor progression occurs in the interval between different imaging studies and/or goes undetected from lack of contrast enhancement. An alternative measure or marker of tumor burden may potentially permit earlier detection of treatment failure and allow for more timely therapeutic changes [6]. Single-molecule markers have been mostly insufficient to properly follow the dynamics of diseases like cancer and should be replaced by multiple marker profiles. The first study that systematically reviewed the diagnostic serum profile in GBM patients was published by Elstner et al. in 2011 and analyzed the serum concentrations of 14 proteins [7]. Nevertheless, no reliable marker has yet been identified for use in the diagnosis and surveillance of patients with malignant gliomas [8].

This review aims to summarize the potential of peripheral blood markers described in the literature and to critically evaluate its applicability to clinical practice.

## 2. Materials and Methods

A thorough search of the literature was performed using the MEDLINE database and Cochrane Reviews (January 2005 through December 2018) using the terms “glioma, serum biomarkers”. Articles in languages other than English were excluded. Only studies on data collected in humans were considered.

We divided the biomarkers into particular categories, although several of them have multiple roles and these categories are highly interrelated: (a) cell growth and vascular proliferation; (b) immune system; (c) inflammation; (d) nutritional status; and (e) coagulation markers. 

## 3. Results and Discussion

The MEDLINE search identified 208 articles from January 2005 through December 2018, using the terms “glioma, serum biomarkers”. Articles in languages other than English were excluded, remaining 200 articles. Only studies on data collected in humans were considered. That gives a total 174 articles. Of these, only 39 articles specifically adressed potential serum biomarkers, 30 prospective studies, 5 retrospective studies and 4 revision studies, corresponding to 52 different molecules and 30 of them are referred only once and were included for final evaluation. Cochrane Reviews did not identify any title. 

The most relevant potential serum biomarkers found in literature are summarized in Table 1. Many of these biomarkers belong to more than one category confirming the interaction between different hallmarks and cancer pathways. Although these biomarkers were closely related to each other, for a better text organization we describe the biomarkers by groups and discuss their potential roles in predicting the presence or the progression of glioblastoma. 

### 3.1. Biomarkers Related to Vascular Proliferation and Cell Growth

Microvascular proliferation is a hallmark feature in glioblastoma. Being a complex process, multiple mechanisms are involved. Several biomarkers were studied in this context. 

*VEGF* is the most important mediator of neovascularization in gliomas [9]. Studies measuring VEGF concentration in the serum of patients with glioblastoma have obtained conflicting results [10]. 

Salven et al. found serum levels of VEGF to be elevated in high-grade brain tumors [11]. In Reynés et al. work, in a series of 40 glioblastoma patients, serum levels of VEGF were twofold higher in patients compared with healthy controls [10]. Only pre-operative samples were collected, so VEGF could not be used as a follow-up biomarker. Stockhammer et al. found no differences between serum VEGF concentration in 6 patients with glioblastoma and in healthy controls [12]. Fine et al. did not find any correlation between serum VEGF levels and tumor response progression, time to progression, or overall survival [13]. Rafat et al. showed that levels of serum VEGF and granulocyte–macrophage colony stimulating factor (*GM-CSF*) are raised in patients with glioblastoma when compared with healthy controls [14]. Due to the lack of consistency in the different studies, some with very few cases, VEGF has not been shown to be a diagnostic or survival marker.

YKL-40 is a 40kDa glycoprotein produced by cancer, inflammatory and stem cells. It probably has a role in cell proliferation and differentiation, inflammation, protection against apoptosis, stimulation of angiogenesis, and regulation of extracellular tissue remodeling [5], and stimulates fibroblasts surrounding the tumor, although in vivo proof of these hypotheses is yet to be obtained [3]. Elevated serum YKL-40 is found in a subgroup of patients with different types of solid tumors [3]. Plasma levels of YKL-40 are highly correlated with age [5], and as plasma YKL-40 increases with age, levels in patients before or during treatment should be evaluated in comparison with age-stratified or age-adjusted reference levels [15]. YKL-40 is over-expressed in glioma tissues but is not expressed in normal brain or normal human astrocytes, suggesting that it is related to glioma initiation and progression [16] and may promote radiation and apoptosis resistance, increased invasiveness, and increased 72 KDa metalloproteinase activity [17]. It was first described as a potential serum biomarker in glioblastoma in 2002 by Tanwar [6], who found that the YKL-40 protein was dramatically elevated in a subset of the GBMs, and essentially below the detection limit for Western blot analysis, in both lower-grade tumors and normal brain extract. Statistically significant differences were detected between YKL-40 serum levels in patients with glioblastoma compared with controls and compared with patients with lower-grade gliomas, as well as between lower-grade tumors compared with controls but no significant differences were detected between high versus low tumor burden in GBM patients. In the series of Hormigo et al., tumor resection resulted in transiently increased serum levels of YKL-40 and reached a peak 24 h after resection [18]. These authors also found a significant difference in YKL-40 values for GBM patients with a complete resection compared with patients with evidence of disease. There was a significant association between the actual value of serum YKL-40 and survival. A low absolute YKL-40 value identified a subset of patients that is more likely to do well. Those patients with persistently normal values had a longer survival and disease-free interval [18]. In the same line of evidence, Bernardi et al. showed that YKL-40 serum levels determined at the 1st week after surgery may be a biomarker of outcome for high-grade gliomas. Despite a wide range of variability in YKL-40 levels, serum levels are higher in GBM patients than in healthy subjects, and surgery has a paramount influence on postoperative YKL-40 serum levels, raising the hypothesis that it may be a putative biochemical marker of extent of tumor resection [19]. Different data was showed by Linde et al. who found very little variation in YKL-40 protein concentrations in serum of patients throughout the disease course [20], and did not observe any correlation between serum YKL-40 and extent of resection or tumor volume. A possible explanation is the short follow-up and the time points of the serum collection. Besides that, no pre-operative values were collected and there were no correlations between these two data. There was no age adjustment nor correlation with tumor tissue expression. In spite of this data, Johansen conclude that there is still insufficient evidence to support its value outside of clinical trials as a screening tool, prognosticator of survival, predictor of treatment response and as a monitoring tool in the routine management of individual patients with cancer [21]. However, YKL-40 is one of the serum biomarkers with more evidence from different groups in GBM, and more robust further studies, with a larger number of patients with better characterization and new quantification techniques will be able to clarify and establish YKL-40 as a true clinically relevant GBM biomarker.

Epidermal growth factor receptor (*EGFR*) is a transmembrane cell surface receptor and a member of the c-erb-B family of tyrosine kinases, known to be overexpressed in a variety of human malignant tumors [22]. *EGFR* pathway plays prominent roles in regulating cell growth, apoptosis, and differentiation. Aberrant *EGFR* signaling may ultimately affect major hallmarks of cancer, including tumor growth, invasion, malignancy, and prognosis [23]. Induction of EGFR expression might be involved in the malignant transformation of human brain cells [24]. 

Choi et al. found that an overexpression of *EGFR* appears to correlate with glioma grade, which is observed in about 40–50% of GBMs compared to 10–26% of anaplastic astrocytomas [25]. *EGFR* gene is amplified in approximately 30–50% of malignant gliomas [26,27]. In the literature, results for the influence of *EGFR* expression on survival according to tissue immunohistochemical staining differ among researchers [25], despite the majority of papers showing a worse prognostic in patients with overexpression of *EGFR*. *EGFR* expression is also correlated with a poor response to radiation therapy [28]. While these results are related to tumor tissue expression, the associations between *EGFR* expression in tissue samples and in blood samples are yet to be demonstrated. Quaranta et al. showed the mean serum EGFR level in brain cancer patients is significantly elevated compared with that of healthy controls, with 73.8% of the patients demonstrating an elevated serum level of *EGFR* [26]. They concluded that serum *EGFR* extracellular domain may be potentially useful as a biological marker of gliomas for prediction of prognosis and follow-up after treatment.

GFAP is a 55kDa intermediate filament protein that is highly expressed in astrocytes as part of cytoskeleton [29] and may serve as an organ-specific marker [30]. The first description of serum GFAP in primary brain tumors patients was made by Brommeland et al. in 2007, including 27 GBM and four anaplastic astrocytoma patients. In a multivariate linear regression analysis, the only significant association was between pre-operative GFAP levels and tumor size. No difference was found between glioblastomas and astrocytomas [30], but the sample size was very small to allow a definitive conclusion. Although putting the possibility that GFAP values may reflect the evolution of the disease or the response to treatments, this extrapolation cannot be done because only the preoperative values were evaluated. Jung et al. studied a group of 50 patients with glioblastoma and found higher levels of serum GFAP compared to non-GBM tumor patients and healthy controls. In addition, serum GFAP concentration was closely linked with GBM tumor volume and tumor necrosis volume. As no follow-up assessment of serum GFAP was done, the only conclusion was that serum GFAP could be a diagnostic biomarker of glioblastoma [31]. Still, the number of patients was too small (n = 50), and in 25% of GBM patients, no detectable levels of serum GFAP were found. Ilhan-Mutlu et al. found that GFAP plasma detectability was strongly associated with diagnosis of GBM in a series of 105 brain tumor patients, but it was only present in 38% of GBM patients (13/34). In these patients, no correlations were found between serum GFAP and tumor volume or survival [32]. It is another small series to allow reliable results. Husain H et al. evaluated the plasma GFAP levels before and after tumor debulking in a series of 33 gliomas. Detectable levels were found in 52% of patients pre-operatively and in 96% of patients post-operatively. In the GBM sub-group (nine patients), three having lower levels and two having higher levels was the main difference to the non-GBM group. Although they found a correlation with tumor grade and serum GFAP levels, the few number of patients enrolled again prevents reliable conclusions [29]. In a more recent prospective study of 33 GBM cases, 14 had elevated GFAP serum concentrations prior to any intervention [33]. Following surgery, all positive patients showed decreasing serum concentrations. An interesting factor was that 16 patients (48%) only did a stereotactic biopsy without reducing the tumor burden. There was no plausible explanation for this. Vietheer et al. concluded that serum GFAP at diagnosis are not predictive for survival or tumor recurrence. 

An important bias is the small number of patients in each study and in the set of studies, precluding solid conclusions about the diagnostic, prognostic and follow-up relevance of GFAP in GBM.

Thrombospondin-1 (TSP-1) has tumor-inhibitory properties and inhibits tumor angiogenesis, however, no difference was found in serum samples between glioma patients and healthy controls [10]. Elstner et al. found a significant difference in serum protein concentration of TSP-1 between patients who survived more or less than 15 months [7]. Analysis of TSP1 alone predicts the survival chance for 80% of the GBM patients, but in conjunction with HSP70 and IGFBP3 the predictability increased to 100% [7]. Reynés et al. did not find any correlation with prognosis and survival. No further studies have been conducted with serum TSP-1 in the glioma context. Few studies and controversial results make it difficult to conclude the relevance of this putative biomarker.

Osteopontin (OPN) is a multifunctional arginine-glycineaspartate (RGD)-containing glycophosphoprotein with cytokine and chemokine properties [34] and has been implicated in tumor progression of a variety of systemic cancers [35]. Expression levels of osteopontin have been correlated with malignancy in human glioma cells and are associated with astrocytoma progression [36]. High cytoplasmic OPN expression in glioblastomas was associated with poor patients’ survival [37]. Sreekanthreddy et al. carried out a detailed analysis of OPN at RNA and protein levels in tumor tissue and serum in different grades of astrocytoma as well as its effect on patient survival [38]. Comparisons of serum osteopontin levels were significant between GBM and normal controls, GBM and anaplastic astrocitomas, and GBM and diffuse astrocytomas. In a set of 30 newly diagnosed GBM patients they found that serum OPN levels > 20 ng/mL significantly correlate with the survival. 

Recoverin (calcium-binding protein p26) is a cytoplasmic calcium-binding protein. The highest levels of expression are seen in the vitreous humor of the eye, but it can also be detected at low levels in the serum of normal adults [8]. Thirkill et al. first identified recoverin as a protein that binds antibodies expressed in patients with cancer-associated retinopathy (CAR), a paraneoplastic syndrome [39]. Sampath et al. found that serum recoverin levels were increased 10-fold in patients with active recurrent GBM compared with normal controls, and patients with quiescent GBM have only a slight increase [8]. The authors concluded that recoverin may be a useful glioma tumor marker, especially for recurrent active disease [8]. The sample only includes 5 patients with active recurrent disease and 2 with no signs of residual or recurrent disease so these conclusions are no more than extemporaneous, and no further studies could definitively confirm these findings. Manley et al. also investigated pediatric patients with glial tumors, and even though there was a correlation with progressive disease and elevated recoverin (A-PROTEIN) levels, the sensitivity and specificity of recoverin as a predictor of disease progression was insufficient to warrant its clinical use [40].

Cathepsin D may be involved in several actions that facilitate tumor progression [41]. Cathepsin D is expressed widely in the brain [42] and was confirmed to be upregulated in high-grade glioma [43]. The high expression of cathepsin D is one of the common features in the tissue samples of the high-grade astrocytomas [41]. Fukuda et al. analyzed the correlation between the molecular information and the clinical data of 78 glioma patients, including tumor grade, invasive nature and survival time. The serum levels of cathepsin D protein were compared between nine patients with low-grade gliomas and 11 patients with high-grade gliomas. The values were significantly higher in high-grade gliomas than in the low-grade tumors [41]. These data suggested that serum cathepsin D may be a potential indicator of the biological aggressiveness of intracerebral gliomas [41], but again, the very small number of patients is not reliable for definitive conclusions. 

Cytokines are a group of multi-functional signaling proteins linked to angiogenesis, cell-growth, stem cell differentiation and regulation of immune responses, including tumor immune surveillance and tumor-induced immunosuppression [44]. In a group of 55 brain tumor patients, including 14 malignant, 17 benign and 24 metastatic Loh J-K show that pre-operative TGF-β1 plasma levels were higher than in the control group. They also found significant decreases in TGF- β1 level in benign and malignant tumors after removal. A slight but significant increase was seen in the metastatic group after surgery. These data allow the distinction between primary and metastatic tumors, but revealed no differences between the different primary tumors, in addition to only 6 being glioblastomas [45].

Bone morphogenetic proteins (BMPs) are members of the transforming growth factor-β (TGF-β) superfamily. The role of BMP2 in glioblastoma has not yet been elucidated [7], but appears to inhibit growth in gliomas [46], and is linked to angiogenesis and stem cell differentiation. BMP-2 expression in tumor tissue was significantly associated with prognosis [47]. Elstner studied the potential role of 14 serum secreted proteins, including BMP2 in a series of 23 GBM patients and 12 control subjects and concluded that the presence of a glioblastoma was only distinguished by serum concentrations of BMP2, CXCL10 and HSP70 in conjunction [7]. Nijaguna M, in a series of 148 GBM patients and 26 healthy controls, found a set of 18 cytokines to be different between the two groups [48]. Only pre-operative samples were collected, which prevents its use as a prognostic or follow-up marker. Schwartzbaum et al. analyzed serum samples from 487 subjects who were subsequently diagnosed with glioma [44]. This was the first study trying to find prediagnostic markers related to glioma risk or to the presence of a preclinical tumor. They have identified five serum cytokines and a cytokine-soluble receptor interaction, each associated with changes in the preclinical risk of glioma, but the authors themselves described some important bias and the results should be evaluated very carefully. These biases are transversal to this type of studies and are related to short follow-up times and small series of patients, with the inherent statistical limitations and implications in the results.

Peripheral IL-8 secretion correlates with its levels in astrocytic tumor tissues [49]. Ilhan-Mutlu et al. found an inverse association of IL-8 plasma concentration with tumor grade in glioma [32]. Elstner et al. show a reduced serum concentration for CXCL10 in glioblastoma patients but they did not find any correlation with the prognosis, tumor size or survival [7]. In a proteomic study with 35 GBM patients and 30 healthy controls, Popescu et al. found three candidate biomarkers for GBM diagnosis, CXCL4, S100A8 and S100A9 [50], but altered levels of these markers were also found in a set of different pathologies and were not specific to GBM.

MMP-9 is the most common metalloproteinase associated with neovascularization of tumors [51]. Expression of MMP-9 has been detected both in tumor and endothelial cells and is overexpressed in glioma. It could play an important role in promoting cell invasion into the surrounding brain and in glioblastoma resistance to antiangiogenic treatment [52]. Raithatha found that increasing levels of MMP-9 in glioma specimens correlates with higher tumor grade [53]. This correlation was also investigated in peripheral blood [18,54]. Hormigo et al. found a significant difference in MMP-9 values for GBM patients with a complete response compared with GBM patients with radiographic evidence of tumor in a series of 30 patients. However, the authors did not find correlations with tumor grade or overall survival [18]. Iwamoto el al. from the same group, and some years later, did not find any correlation with serum MMP-9 levels and tumor size, survival or chemotherapy response, concluding that serum MMP-9 showed no utility in determining glioma disease status in a cohort of 111 GBM patients, and was not a clinically relevant prognostic marker of survival [54]. Ricci S et al. tested a series of 60 brain tumors (20 gliomas, 20 meningiomas and 20 metastases) and 25 controls and showed that MMP-9 is significantly increased in the sera from patients with CNS tumors compared to healthy controls [55]. Again, this study had a very small number of patients of each tumor type to allow solid conclusions.

Tissue Inhibitor of Metalloproteinase-1 (TIMP-1) is a glycoprotein that is ubiquitously expressed in numerous human cells and tissues [56], with a wide spectrum of functions. Crocker data from 36 newly diagnosed GBM patients and five healthy controls showed that, in glioblastoma patients, a higher serum TIMP-1 level at presentation predicts shorter survival, and that TIMP-1 levels in the glioblastoma patients are significantly higher than in normal controls [57], suggesting that serum TIMP-1 levels are an independent predictor of survival. Aaberg-Jessen et al. in a cohort of 20 GBM patients did not find significant differences between plasma TIMP-1 levels and heathy control subjects [58]. They did not find any correlation between tissue levels and plasma levels, concluding that scoring TIMP-1 immunoreactivity is a better choice than measuring the plasma levels.

### 3.2. Biomarkers Related to Immune System

Immunologic factors have long been hypothesized to play a role in glioma risk, and secretion of immunosuppressant molecules by high-grade gliomas is well documented [59]. Some epidemiological studies have reported that adults with glioma are 1.5 to 4-fold less likely than controls to report a variety of allergies [60], supporting an inverse association between self-reported history of allergies and the risk of glioma. Indeed, a meta-analysis showed that glioma risk was reduced by 39% in people with a history of allergies compared with people with no history of allergies [61].

IgE is the class of antibody most responsible for atopic allergic diseases [62] and is a CD23 ligand [63]. An antitumor role for IgE has been proposed for solid tumors [64,65]. Wrensch et al. studied the serum IgE levels in a series of 115 GBM patients, showing that those with elevated levels had nine months longer survival than those with normal or borderline IgE levels [66]. Lin Yi et al. compared plasma IgE levels between different grades of gliomas in a series of 252 patients and 25 healthy controls and found that plasma IgE levels in low-grade glioma patients were lower than in high-grade glioma patients. No significant differences were found between grade III astrocytomas and GBM. For patients whose IgE increased significantly (increase > 100 ng/mL), the survival time was significantly longer than those patients whose IgE level did not increase significantly (127.5 ± 13.4 weeks vs. 62.3 ± 12.4 weeks). They found an increase of IgE levels in 24 patients after tumor treatment [65]. Calboli et al. analyzed data collected from four independent cohorts comprising 169 glioma cases and 520 controls and did not observe a statistically significant association between total IgE level and risk of glioma. A more careful examination revealed a lower risk for “borderline elevated” total IgE levels (25–100 kU/L) than with “elevated” total IgE levels (>100 kU/L), compared with clinically normal total IgE levels (<25 kU/L) [67]. Schlehofer B et al. in a case-control study found that the risk of glioma was inversely related to allergic sensitization and concluded that individuals with allergic sensitization are at reduced risk of developing glioma [68]. Schwartzbaum et al. conducted the largest study concerning the levels of IgE and the glioma risk and found that elevated levels of prediagnostic allergen-specific IgE and total IgE are associated with reduced risk of both glioblastoma and glioma, but the association between testing positive for allergen-specific IgE and decreased tumor risk was restricted to the female gender [44].

Despite some findings from most studies are conflicting, and more data should be obtained, it seems that there is an important correlation between IgE and GBM.

CD14 is an important component of the innate immune Toll-like receptor system [59] and CD23 is a mediator of the allergic response, exhibits proinflammatory properties and can function to enhance antigen presentation of IgE antigen complexes [59] and regulate IgE production [69]. In a case-control study of 1078 cases for CD14, 1064 cases for CD23 and 736 controls, comprised 57 GBM patients among other malignant brain tumors, CD14 accumulated in GBM but not among the low-grade astrocytomas, in contrast to sCD23, which means serum levels was lower for glioma cases than controls [59]. Some reports suggest that elevated CD23, either on neoplastic cell surfaces or as a soluble form, is a useful marker in either diagnosis or prognosis of disease [63] and sCD23 levels could be used as a marker of glioma risk.

### 3.3. Biomarkers Related to Inflammation

Chronic inflammation increases the risk of developing some types of cancer and the inflammatory cell-driven microenvironment might lead to tumor initiation and promotion [10]. Several inflammatory markers were associated with the presence or progression of glioblastoma, and lymphocytic infiltration is a common finding.

Lopes M et al. studied the influence of neutrophil–lymphocyte ratio (NLR) in prognosis in a series of 140 GBM patients. They did not find NLR to be an independent poor prognostic factor in the total cohort, but in the subgroup of patients who completed the Stupp protocol, a preoperative NLR > 7 correlated with shorter overall survival [70]. These results do not corroborate the previous findings of Bambury et al. and of Han et al. showing a significant correlation between NLR > 4 and a shorter overall survival [71,72].

IL-6 amplification is also involved in angiogenesis and tumor progression, and although it was associated with shorter survival in glioblastoma patients [73], Reynés et al., in a series of 40 GBM patients and 60 healthy controls, found that all inflammation markers studied, including IL-6, were significantly elevated in glioblastoma patients compared with controls [10]. Shan Y et al. studied a series of 16 healthy controls and 86 glioma patients, 43 of which were GBM [74], and found IL-6 in the serum of glioma patients increased as compared to the control group, and this increase was related to the grade of glioma and reduced markedly after surgery. These decreases were closely related to the prognosis of glioma patients and could be used for prediction of clinical prognosis [74].

Amyloid A1 (SAA1) is a sensitive acute phase reactant primarily produced by the liver in response to acute inflammation and affects proliferation, migration and invasion of glioblastoma cell lines [75]. There is evidence that elevated serum levels of SAA correlate directly with poor prognosis in various systemic cancers such as lung, breast, melanoma, gastric, prostate, esophageal, endometrial and renal [75].

Knebel et al. in a series of 148 astrocytoma and 10 non-neoplastic patients showed that patients with astrocytic tumors have higher SAA serum concentrations than the non-neoplastic controls. However, they did not find statistically significant differences among the different astrocytoma grades [75]. The SAA produced by glioblastoma did not contribute to the increase in serum SAA, rather this high serum concentration is due to liver production as part as an inflammatory response to the tumor. They found no association between SAA serum levels and patient survival.

Alpha 2-Heremans-Schmid glycoprotein (AHSG) is a 64-kDa serum protein, a liver-derived circulating protein and is a ligand for tyrosine kinase receptors [76]. AHSG is a negative acute-phase protein. The serum AHSG concentration is decreased in individuals with infection, malignancies, liver diseases, malnutrition and end-stage renal disease [77]. It also has a link between obesity, fatty liver, insulin resistance, and metabolic syndromes [78].

There are two studies concerning AHSG and brain tumors. Ribom et al., in a 2003, paper showed an increase in CSF levels in patients with low-grade gliomas [79]. Petrik et al. first determined AHSG as a survival predictor in GBM and then, in an additional series of 72 GBM patients, validated its prognostic value. They demonstrated that low serum alpha 2-HS glycoprotein concentration correlates with increased tumor proliferation. However, reduced serum AHSG is not a specific biomarker of GBM. It is also low in patients with non-glioma brain pathologies and in patients with non-CNS tumors [80].

Heat shock 70-kDa protein is a heat shock protein (HSP), which is a group of phylogenetically conserved proteins found in all prokaryotic and eukaryotic cells [81]. Increased HSP70 antibody levels have been found in the serum of lung cancer patients, indicating that it may be a biomarker for several tumor types [7]. Different patterns of HSP expression seem not to account for the differential response of these tumors to adjuvant cytotoxic therapy [82].

Glioblastoma patients have an increase serum concentration of HSP70 compared with normal controls but in the Elstner study, HSP70 was only reliable when associated with BMP2 and CXCL10 [7]. No additional evidence has been reported to support the association between HSP70 and glioma, making this protein a weak biomarker for prognosis and survival.

### 3.4. Biomarkers Related to Nutritional Status

The nutritional status has been studied in many cancers, being a prognostic factor. The association between nutritional status and inflammatory status may play a role in the development of glioblastoma and seems to be an important prognostic factor. The biomarkers described to be related to glioblastoma are albumin and IGFBP-2.

Serum albumin measures long-standing malnutrition and is also associated with systemic inflammation.

Borg et al. analyzed a series of 549 GBM patients with pre-operative albumin assayed. They showed significantly higher perioperative mortality (≤30 days post-operatively) in the hypoalbuminemia group, which also showed significantly lower conditional survival after 30 days. They could not find a definite reason but correlated indirectly to an inflammatory status mediated by cytokines TNFα and IL-6 [83].

Han et al. showed that patients with low serum albumin levels (<30 g/L) had a significantly shorter overall survival than those with levels in the normal range (median 6.0 vs. 15.0 months) and this is partial due to the nutritional status of patients but mostly to the inflammatory response and higher IGFBP-2 levels [72].

The prognostic nutritional index (PNI), which was calculated from the following formula: 10 × serum albumin (g/dL) + 0.005 × total lymphocyte count (per·mm^3^), reflected the nutritional and immune status of patients with cancer [84].

The preoperative albumin-to-globulin ratio (AGR) could reflect both malnutrition and systemic inflammation in cancer patients. In Liu study, patients with low preoperative albumin-to-globulin ratio have a better prognosis and conclude that preoperative AGR was significantly associated with OS in GBM patients [85].

The possible mechanism by which PNI is involved with tumor prognosis may be that a low serum albumin level deteriorates the general nutritional status of the patients [84].

He et al. created a cumulative score using serum albumin plus plasma fibrinogen. Fibrinogen was shown to play important roles both in tumor cell proliferation, migration and angiogenesis and as regulator of inflammation in solid tumors. They conclude that higher pretreatment FA score was an independent prognostic factor of PFS and OS of patients with high-grade gliomas and was significantly associated with advanced age and higher tumor grade [86].

### 3.5. Biomarkers Related to Coagulation

Thrombin expression is involved in brain development, protection and regeneration. Besides its role in hemostasis, thrombin facilitates receptor-mediated inflammatory responses, apoptosis, cell proliferation-/modulation and angiogenesis, and is involved in complex signaling pathways in glioblastoma [87].

Reynés G et al. found that prothrombin fragments 1 + 2 and endogen thrombin generation levels were significantly higher in glioblastoma patients than in controls [10]. No further studies associated serum expression of coagulation markers and glioblastoma, even though these factors have a preponderant role in inflammation and gliomagenesis.

### 3.6. Circulating Tumor DNA

Circulating tumor DNA (ctDNA) has been the subject of investigations in the last few years. Despite the technical challenges and the very low concentrations of nucleic acid in biofluids, the information obtained is of paramount importance [88]. In normal individual and in early-stage cancers the ctDNA concentrations are very low, but these levels significantly increase with tumor progression and there is growing evidence that ctDNA concentrations are related to tumor burden, cellular turnover, cancer stage, and therapy response [89]. ctDNA could be obtained regularly over time, reflecting the real composition, the tumoral heterogeneity and the evolution of the tumor throughout time [90]. ctDNA allows us to overcome some difficulties of the magnetic resonance imaging (MRI) in the follow-up of the patients. Treatment effects (pseudoprogression), progressive disease and radiation necrosis are not always easy to distinguish in advanced brain imaging modalities or metabolic imaging such as PET scan [89].

Piccioni et al. studied 665 blood samples from 419 consecutive patients with primary brain tumors. Somatic alterations were detected in 302 samples and in 55% of GBM patients. One-quarter of samples had a ctDNA alteration detected that suggested eligibility for an off-label targeted therapy regimen and almost half of patients had a ctDNA alteration detected that suggested eligibility for a targeted therapy clinical trial [91]. Saenz-Antonanzas et al. concluded that ctDNA has great potential to identify biomarkers for diagnosis, prognosis, and therapeutic response and allow genotype-directed therapies and personalized clinical management of glioblastoma patients [90].

## 4. Conclusions

The pivotal criterion with regard to the potential clinical value of a candidate cancer biomarker is the consistency and strength of the association between the biomarker and the outcome or disease of interest, and the extent to which it is an improvement on either adding to or replacing established tools [5]. Of the various putative GBM biomarkers, YKL-40 emerges as particularly promising in various studies, suggesting it may be useful as a prognostic predictor and may have a role in screening and monitoring of GBM patients.

Although a significant activation of inflammation, angiogenesis and coagulation processes have been demonstrated in patients with glioblastoma, none of the reported circulating markers could be associated with clinical outcome.

Single molecular markers are mostly insufficient to follow the dynamics of diseases such as cancer and will be replaced by multiple marker profiles^.^ When in conjunction, the serum concentrations of certain proteins may indicate the presence of a glioblastoma with high sensitivity and specificity.

Despite these findings, none of these proteins alone was sufficiently specific and sensitive to serve as a diagnostic marker. It is important to note that none of these markers have been implemented in the clinical routine and so do not have clinical value. We can speculate that this lack of consistent results is due to the disease and patient heterogeneity. Another explanation is the fact that some studies have a reduced number of patients, lack of standardization in the sample collection, different quantification methods and lack of results validation. Most studies are small retrospective series that do not allow robust statistical conclusions. The methodology used varies depending on the biomarker studied, with no uniform criteria allowing homogenization of the results. The cut-offs used are different in various studies. The follow-up period is short. Many of the biomarkers were only collected preoperatively, with no longitudinal assessment over the course of the disease. Most markers are involved in various physiological and pathological processes and are not specific to glioblastomas, contradicting the definition of biomarker. The concomitance of other pathologies with increased expression of the same biomarkers was not excluded in most studies. Another important limitation is that a subset of patients with GBM have normal serum levels of these markers.

In the complex context of gliomagenesis, progression, and regrowth, with a multifactorial network involving vascular proliferation, cell growth, inflammatory, immunological, coagulation and nutritional factors, trying to address only one factor that could function as a biomarker of diagnosis, prognosis or disease status could be impossible and only a set of biomarkers can actually give appropriate and more accurate information. However, at the moment, this is not possible given the length of the process and the costs involved, which makes it inappropriate for each patient per se.

We conclude that although these markers may be useful in identifying gliomas, they lack enough acuity to avoid biopsy and tissue analysis. This is the reason why there is currently no true blood biomarker specific to glioblastomas. In the future, a multi-biomarker panel signature may prove to be a very useful tool in glioma subtypes diagnosis, improving prognosis information and treatment response prediction. Single-cell analyses, ctDNA or circulating tumor cells analyses are promising technologies that may improve diagnostic accuracy and quality of follow-up in GBM patients. In this perspective, it is our conviction that research must be consolidated by prospective studies targeting routine, low-cost, disease-specific biomarkers allowing early diagnosis, better prognosis stratification and response prediction. Additionally, the characterization of a panel of genetic alterations in glioblastoma specimens that match peripheral blood markers, may increase the sensitivity of the screening biomarkers. Our main effort at this point is focused on ctDNA, namely in identifying genomic alterations that may allow a more efficient follow-up and possible targeted therapies, matching off-label and clinical trials agents.

## Figures and Tables

**Table 1:** Serum biomarkers reported in the literature with potential application in glioblastoma.

Vascular Proliferation	Cell Growth	Inflammatory	Immune System	Coagulation	Nutritional
BMP-2	α2-HS glycoprotein	CD23	CD14	Fibrinogen	IGFBP-2
CXCL 10	BMP-2	Albumin	CD23	ETG	Albumin
Haptoglobin α2	Cathepsin D	Ratio N-L	IgE	Prothrombin factors	
Metalloproteinase 9	CXCL10	Ratio P-L	Osteopontin	TF (Tissue factor)	
Osteopontin	EGFR	Amyloid A1	Recoverin		
YKL-40	GFAP		TGF-β		
VEGF	Metalloproteinase 9		TNF-α		
PDGF-b	Osteopontin		IL-6		
TGF-β	Recoverin				
TNF-α	PDGF-b				
TSP1	IGF-1				
	TGF-β				
	TNF-α				
	IL-8				
